# Augmenting perceived stickiness of physical objects through tactile feedback after finger lift-off

**DOI:** 10.3389/frobt.2024.1415464

**Published:** 2024-09-18

**Authors:** Tadatoshi Kurogi, Yuki Inoue, Takeshi Fujiwara, Kouta Minamizawa

**Affiliations:** ^1^ Toyoda Gosei Co., Ltd., Aichi, Japan; ^2^ Keio University Graduate School of Media Design, Yokohama, Japan; ^3^ Graduate School of Informatics and Engineering, The University of Electro-Communications, Tokyo, Japan

**Keywords:** haptic augmented reality, stickiness, adhesion-separation contact mode, dielectric elastomer actuator, tactile feedback

## Abstract

Haptic Augmented Reality (HAR) is a method that actively modulates the perceived haptics of physical objects by presenting additional haptic feedback using a haptic display. However, most of the proposed HAR research focuses on modifying the hardness, softness, roughness, smoothness, friction, and surface shape of physical objects. In this paper, we propose an approach to augment the perceived stickiness of a physical object by presenting additional tactile feedback at a particular time after the finger lifts off from the physical object using a thin and soft tactile display suitable for HAR. To demonstrate this concept, we constructed a thin and soft tactile display using a Dielectric Elastomer Actuator suitable for HAR. We then conducted two experiments to validate the effectiveness of the proposed approach. In Experiment 1, we showed that the developed tactile display can augment the perceived stickiness of physical objects by presenting additional tactile feedback at appropriate times. In Experiment 2, we investigated the stickiness experience obtained by our proposed approach and showed that the realism of the stickiness experience and the harmony between the physical object and the additional tactile feedback are affected by the frequency and presentation timing of the tactile feedback. Our proposed approach is expected to contribute to the development of new applications not only in HAR, but also in Virtual Reality, Mixed Reality, and other domains using haptic displays.

## 1 Introduction

One of the primary goals for researchers involved in haptic presentation is to develop a method that actively replicates all haptic sensations at a level where users feel as if they are touching real objects. Despite various innovative haptic presentation methods proposed, only a limited range of haptic sensations can be reproduced with such high fidelity that users feel as if they are interacting with real objects. This limitation arises from the complexity of human haptic perception, which is influenced by multiple physical factors such as shape, hardness, roughness, friction, warmth, and coldness. Reproducing all these factors convincingly using only haptic displays poses significant challenges. Haptic Augmented Reality (HAR), which overlays haptic stimuli generated by physical objects onto haptic feedback produced by haptic displays (Jeon et al., 2015), holds promise in addressing this challenge. HAR compensates for the limitations of haptic displays by integrating haptic feedback from physical objects with active haptic feedback from the display itself. For instance, in developing a tactile presentation system that actively modulates roughness, HAR could focus on reproducing only the roughness information using tactile displays while supplementing other passive haptic elements (such as shape, hardness, and temperature) using physical objects. Thus, HAR has the potential to offer a wide range of highly realistic haptic experiences and is expected to enhance user experiences across various applications, e.g., tumor palpation training (Jeon et al., 2011; Jeon and Harders, 2014), surgical training (Fani et al., 2017), task performance enhancement (Borst and Volz, 2005; Maisto et al., 2017), prototyping assistance (Withana et al., 2018), and entertainment (Lopes et al., 2018).

Haptic experiences occur so often in healthcare and medicine. For instance, doctors may feel the hardness of tumors by palpating affected areas, or feel stickiness when touching oily or sweaty skin that appears as one of the symptoms of skin diseases (Gerling and Thomas, 2005; Schwartz et al., 2006; Swinn et al., 2003). In particular, the stickiness of skin is a sensation relevant not only in medical contexts but also in daily healthcare, such as when users check the health of their skin or the effectiveness of cosmetics products. Several studies have examined the perception mechanism of the stickiness sensation, revealing two contact modes: friction, which occurs horizontally on the skin surface when tracing an object horizontally, and adhesion-separation, which occurs when an object attached to the skin separates vertically from the skin as the finger lifts off from the object (Jiang and Wang, 2020; Kim et al., 2017; Yeon et al., 2017). These two contact modes were suggested to exhibit different characteristics (Jiang and Wang, 2020) and be indicated to be selectively employed depending on the task, cultural influences, and other factors (Arakawa et al., 2021).

Nonetheless, most prior approaches to modulating stickiness in HAR have primarily concentrated on the friction contact mode, which occurs when a finger moves horizontally over object surfaces. For instance, (Winfield et al., 2007), proposed a method to reduce physical object friction without the need for attaching a tactile device to the fingertip by utilizing the squeeze film effect. (Bau et al., 2010). proposed a method to enhance the frictional force between a physical object surface and a finger by using the electrovibration caused by the electrostatic attraction of the finger to the surface. (Salazar et al., 2020). demonstrated that the stiffness, shape, and friction of a physical object can be modulated by employing a wearable cutaneous stimuli display. These previous findings enabled stickiness modulation by altering the friction force during horizontal movement on the object surface. However, the friction contact mode and the adhesion-separation contact mode involve distinct directions and temporal variations of the forces acting on the finger. Thus, these methods, which do not prioritize the accurate replication of the force exerted when a finger is vertically pulled away from the adhering object, cannot effectively modulate the adhesion-separation contact mode.

Therefore, we propose a method to present the sensation of stickiness in the adhesion-separation contact mode in HAR. A key challenge in accomplishing this is the difficulty of thin and soft tactile displays designed for HAR to accurately reproduce the vertical pulling force exerted by adhesion on the skin of the finger. Several haptic displays capable of simulating adhesion in the adhesion-separation contact mode have been proposed in Virtual Reality (VR) and other related fields. For instance, (Yamaoka et al., 2008), utilized a suction mechanism to simulate the force of adhesion pulling on a finger, thus generating a sensation of stickiness. (Hashimoto et al., 2009). reported that the perception of stickiness could be elicited by applying negative pressure to the palm using a cone-shaped loudspeaker. (Ishihara et al., 2020). introduced a technique for actively modulating physical stickiness by employing a temperature-dependent variable sticky substance. However, these methods typically necessitate covering the finger with bulky haptic displays to simulate suction or adhesion, thereby hindering the conveyance of detailed tactile sensations from the physical object to the user’s finger. To address this limitation, we focused on the mechanism of human sticky perception in the adhesion-separation contact mode.

**FIGURE 1:**
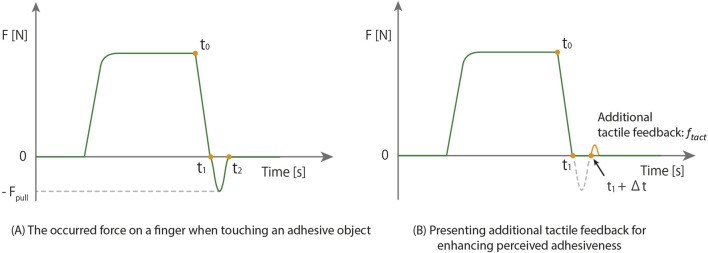
The working principle of a basic dielectric elastomer actuator and developed DEA-based tactile display. **(A)** the occurred force on a finger when touching an adhesive object. **(B)** presenting additional tactile feedback for enhancing perceived adhesiveness..

Several studies exploring the relationship between perceived sticky in this mode and physical variables between skin and object surfaces revealed that the intensity of perceived sticky is not dependent on the force exerted when the finger is lifted off from the attached object ( $F_{\mathrm{pull}}$ in Figure 1), but rather on the duration required for the attached object to completely detach from the skin ( $t_{2}-t_{1}$ in Figure 1) (Mith et al., 2008; Nam et al., 2020). The force exerted on the fingertip in the adhesion-separation contact mode can be decomposed into several different time intervals, as shown in Figure 1A. $t_{0}$ is defined as the time when the pressing force begins to decrease after the finger presses the object. $t_{1}$ is the time when the force exerted on the fingertip is closest to 0 N while the finger is being separated from the object. $t_{2}$ is defined as the time when the finger is completely separated from the object for the first time. If the object is sticky, a negative force $-F_{\mathrm{pull}}$ , is generated between $t_{1}$ and $t_{2}$ , pulling the fingertip due to the object attached to the finger during the separation process. At first glance, this negative force $-F_{\mathrm{pull}}$ , pulling on the fingertip might seem to have a significant effect on the perceived stickiness. However, several studies on the adhesion-separation contact mode have surprisingly shown that this notion is incorrect. Several studies exploring the relationship between perceived sticky in this mode and physical variables between skin and object surfaces revealed that the intensity of perceived sticky is not dependent on the force exerted when the finger is lifted off from the attached object ( $F_{\mathrm{pull}}$ in Figure 1A), but rather on the duration required for the attached object to completely detach from the skin ( $t_{2}-t_{1}$ in Figure 1A) (Mith et al., 2008; Nam et al., 2020). Building upon these findings, we speculated that humans perceive sticky not by a physical adhesive force $F_{\mathrm{pull}}$ , but rather by sensing the complete detachment of the attached object from the skin through tactile cues. Based on this speculation, we hypothesized that stickiness could be perceived by providing additional tactile feedback ( $f_{\mathrm{tact}}$ in Figure 1B) that signifies the complete detachment of the attached object from the skin at a particular time after the finger lifts off from the physical object ( $t_{1}+\Delta t$ in Figure 1B). If this hypothesis proves valid, then a thin-film and flexible tactile display could effectively reproduce stickiness without the need to replicate the continuous tension exerted by the attached object on the skin ( $F_{\mathrm{pull}}$ in Figure 1A). To test this hypothesis, we developed a thin-film and soft tactile display and conducted two user experiments to evaluate perceived stickiness.

The main contributions of our study are summarized as follows:

• We proposed a new method to adjust how sticky physical objects feel using tactile feedback.

• Using a Dielectric Elastomer Actuator (DEA), we built a tactile presentation system for HAR to augment perceived stickiness.

• In experiment 1, we indicated the potential for augmenting stickiness by providing additional tactile feedback after a particular time after the finger lifts off from the physical object.

• We conducted experiment 2, which involved testing multiple tactile feedback types and timings, revealing their impact on the stickiness experience.

The rest of this paper is organized as follows: Section 2 provides a review of related research. Section 3 introduces the tactile display employed for hypothesis testing, covering its structure, performance evaluation, and tactile feedback algorithm. In Section 4, we assess the performance of our stickiness augmentation approach. Section 5 outlines a user study conducted to gain insights into the stickiness experience generated by our approach. Finally, we conclude this paper with a summary of our work in Section 6.

## 2 Related work

### 2.1 Haptic augmented reality

Research in HAR explored various applications, including medical training (Jeon et al., 2011; Jeon and Harders, 2014), surgical procedures (Li et al., 2007; Yao et al., 2004), and workplace assistance (Abbott et al., 2007; Abbott and Okamura, 2003; Nojima et al., 2002; Zoran and Paradiso, 2012). However, these studies assumed human interaction with the environment through tools. Conversely, some HAR systems were designed for direct interaction with objects using the user’s finger, akin to clinician palpation. An essential requirement for such HAR systems is to ensure that fine haptic stimuli from physical objects are effectively transmitted to the user’s finger via the haptic display. To address this, researchers proposed different approaches. One method involves haptic presentation techniques that do not encase the finger with a haptic display, e.g., nail-mounted haptic display (Ando et al., 2002), haptic displays on different body parts (Asano et al., 2014; Salazar et al., 2020), fingerpad deformation-restricted display (Tao et al., 2021), electrical muscle stimulation (Lopes et al., 2018), electrovibration (Bau et al., 2010), reverse electrovibration (Bau and Poupyrev, 2012), and the squeeze film effect (Winfield et al., 2007). Another approach is to utilize thin-film, soft tactile displays to convey detailed haptic stimuli from physical objects to the fingers. Examples of such displays include the Fabric Yielding Display (Condino et al., 2016; Fani et al., 2017), electrotactile stimulation (Kajimoto et al., 2004; Withana et al., 2018; Yoshimoto et al., 2015), and DEA (Ji et al., 2021). A study investigating the impact of device rigidity attached to the skin on environmental haptic stimuli found that lower rigidity, determined by device thickness and elasticity, resulted in reduced reduction in tactile sensitivity (Nittala et al., 2019). Therefore, even when a finger is covered with a tactile display, the use of thin film and soft tactile displays can transmit fine tactile stimuli to the finger.

These methods primarily focused on actively modulating the properties such as hardness, softness, friction, shape of physical objects in HAR. However, they do not focus the modulation of the stickiness sensation arising from the adhesion-separation contact mode, which occurs when the finger vertically lifts off from the physical object. Hence, we propose a HAR method aimed at actively augmenting the stickiness perceived by humans during the adhesion-separation contact mode.

### 2.2 Stickiness haptic presentation method

In VR, some haptic feedback methods have been proposed to reproduce stickiness. While these studies primarily focus on VR rather than HAR, the underlying principles may apply to HAR as well. For instance, (Costes et al., 2019), demonstrated that by pseudo-haptics, which control visual stimuli in response to the user’s finger movements on a touchscreen, it is possible to modulate the perceived stickiness. Although this method primarily targeted interaction on touch screens, thus modulating stickiness in the friction contact mode, its extension to three dimensions suggests potential applicability to the adhesion-separation contact mode. Indeed, (Yem et al., 2018), showed that the perception of stickiness in the adhesion-separation contact mode could be reproduced using pseudo-haptics. Additionally, they demonstrated that stickiness could also be perceived by selectively stimulating the flexor tendons inside the finger through transcutaneous electrical stimulation.

These methods utilizing pseudo-haptics or electrical stimulation of flexor tendons enable the modulation of stickiness in the adhesion-separation contact mode. Furthermore, since there is no need to wear bulky haptic displays on fingertips, these approaches are considered applicable to HAR. However, these approaches rely on proprioceptive senses to induce the perception of stickiness, thus may have limitations on the reproducible stickiness sensation. It has been suggested that pseudo-haptics is an illusion of proprioception that arises from the dominance of visual information when there is a discrepancy between visual input and proprioceptive feedback (Lécuyer, 2009). Therefore, using pseudo-haptics to present stickiness can be regarded as a method of haptic presentation that influences proprioceptive senses. Similarly, transcutaneous electrical stimulation of flexor tendons is a technique that evokes haptic sensations through proprioceptive feedback generated by stimulating the tendons. Consequently, this method can also be considered a method of presenting stickiness by affecting proprioceptive senses, much like pseudo-haptics. Here, proprioceptive senses are less sensitive to stimuli of smaller magnitudes compared to cutaneous sensations. Thus, the contribution of proprioceptive senses to haptic perception decreases as the force on the fingertips diminishes, while the contribution of cutaneous sensations increases (Matsui et al., 2013). Consequently, while proprioceptive-focused stickiness presentation approaches may be suitable for reproducing strong stickiness, they may have limitations in reproducing weak stickiness that is not perceivable through proprioceptive senses alone.

Hence, in this study, we propose a novel approach focusing on stickiness perception derived from cutaneous sensations, aiming to reproduce stickiness perception through the stimulation of tactile sensations.

### 2.3 DEA-based haptic display

**FIGURE 2:**
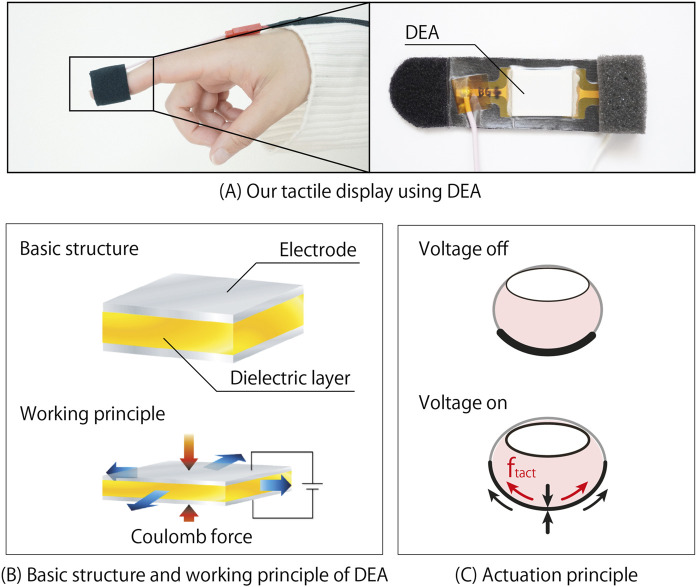
The working principle of a basic dielectric elastomer actuator and developed DEA-based tactile display. **(A)** our tactile display using DEA. **(B)** basic structure and working principle of DEA. **(C)** actuation principle.

The basic DEA comprises two layers of soft electrodes and one layer of soft dielectric material (Pelrine et al., 1997). When a potential difference is applied between the electrodes, the DEA contracts in the stacking direction while stretching in the in-plane direction due to the Coulomb force between the electrodes (Figure 2B). Upon reducing the potential difference, the DEA returns to its initial shape, driven by the restorative force of the material. Consequently, the DEA can produce various vibrations by adjusting the frequency and voltage of the applied voltage. DEAs demonstrate high efficiency in displacement and actuation force relative to their size and weight, despite being thin and lightweight (Gu et al., 2017). For instance, (Ji et al., 2021), achieved a tactile display with a thickness of only 18 μm . Moreover, the soft materials comprising the electrodes and dielectrics make DEAs inherently soft. Therefore, DEAs enable the development of thin and soft haptic displays, facilitating the transmission of detailed haptic sensations from physical objects to the fingers while covering fingers.

Various DEA-based haptic displays proposed, including taxel array tactile displays (Koo et al., 2008), multilayered configurations (Jungmann and Schlaak, 2002), hydrostatic coupling and hydraulic amplification (Carpi et al., 2009), convex protrusion deformation (Koo et al., 2008; Mun et al., 2018), integrated visual-haptic interfaces (Yun et al., 2019), and ultra-thin DEA-based tactile display (Ji et al., 2021). Inspired by these innovations, we developed a thin-film, soft DEA-based tactile display to implement stickiness augmentation method in the adhesion-separation contact mode for HAR.

## 3 System

### 3.1 DEA-based tactile display

Our proposed method for stickiness augmentation in the adhesion-separation contact mode for HAR involves introducing additional tactile feedback to evoke the sensation of complete detachment of the object from the skin, thereby altering the perceived stickiness. In this study, we used vibration as the additional tactile feedback instead of skin stretching or quasi-static indentations. This choice was made because vibration is a common and widely used method for tactile presentation. Identifying a technique that can convey stickiness through vibration feedback could make it broadly applicable, not only to the device used in this study but also to other widely used devices. Figure 2 shows the tactile display developed to assess the effectiveness of this approach. Our tactile display comprises a DEA, a stretchable band with Velcro for finger attachment, a sponge for improved wearability, and a protective film. Users wear this tactile display to their fingertips without applying any voltage to the DEA (Figure 2C). Subsequently, when voltage is applied to the DEA at the appropriate timing, it contracts in the stacking direction and stretches in the in-plane direction. Upon cutting off the voltage, the DEA returns to its initial state, providing tactile stimulation to the finger in three dimensions.

Although our DEA was manufactured with a thickness of only 0.672 mm to transmit detailed tactile stimuli from physical objects to the fingertips, its in-plane size was 10.0 mm × 16.0 mm (length × width). Consequently, the tactile sensation perceived by users is primarily influenced by displacement in the in-plane direction rather than the stacking direction. As such, we mainly focus on skin deformation caused by displacement in the in-plane direction in this paper. The tangential force $f_{\mathrm{tact}}$ exerted on the user’s finger can be calculated using the following Equation 1.

$$f_{\mathrm{tact}}=k_{\mathrm{skin}}\Delta d_{f,V}$$ (1)

$k_{\mathrm{skin}}$ represents the stiffness of the finger, which was determined to be $k_{\mathrm{skin}}$ = 0.53 mN/ μ m as in reference (Biggs and Srinivasan, 2002). Additionally, $\Delta d_{f,V}$ denotes the in-plane displacement of the DEA when a voltage of any frequency is applied. For instance, the maximum $\Delta d_{f,V}$ was measured to be 57.0 μ m when a voltage of 0.5 kV at 10 Hz was applied, as detailed in Section 3.2 below.

For the dielectric layer of our DEA, we utilized a material composed of a blend of silicon and ferromagnetic material (with a relative permittivity of 4 and a Young’s modulus of 0.8 MPa). The electrodes were fabricated from a mixture of carbon black powder and silicon. Each layer of the dielectric material had a thickness of 22.4 μ m, while each electrode layer was 0.15 μ m thick. To enhance the output displacement, we stacked 10 layers of dielectric and electrode materials. This tactile display was inspired by (Kurogi et al., 2019). However, we improved the output by modifying the materials used in the construction of the DEA. Additionally, we enhanced the overall thickness and softness of the tactile display, making it better suited for HAR applications.

### 3.2 Technical evaluation

As for the basic performance assessment of our DEA-based tactile display, we conducted measurements the output displacement characteristics concerning voltage and frequency. While a camera-based method was initially considered for measuring in-plane displacement, it posed limitations in capturing high-frequency vibrations, such as at 250 Hz (Hayashi et al., 2019). Hence, for this evaluation, we employed a displacement meter to measure the displacement in the stacking direction of our tactile display. Subsequently, we derived the in-plane displacement using the correlation between the stacking direction displacement and the in-plane displacement (Pelrine et al., 1997). Note that we assumed our DEA to have a Poisson’s ratio of 0.5 and to be isotropic in the in-plane direction.

To measure the stacking direction displacement, we placed the tactile display between two fixtures and positioned the displacement meter above it. We applied five different voltages ( $V_{pp}$ = 200, 300, 400, 800, and 1000 V), while sweeping the frequency linearly from f = 1Hz to 300 Hz over a duration of 30 s.

**FIGURE 3:**
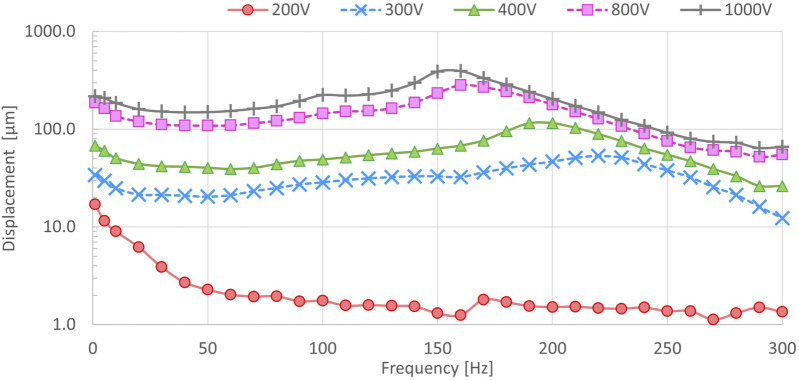
The output displacement of our DEA-based tactile display.

Figure 3 depicts the in-plane displacement calculated from the measurement results. Overall, there was a tendency for displacement to increase with higher applied voltage, although it was observed that the displacement varied depending on the frequency. The variation in displacement with frequency is attributed to factors such as the resonance frequency of our tactile display, the response characteristics of the DEA including viscosity, and the voltage drop associated with high-frequency voltage application. This indicates that the output displacement with our tactile display is influenced by both frequency and voltage. We also prepared over 30 combinations of vibration feedback with various frequencies and voltages using our developed tactile display to preliminary internal explore perceivable frequency and voltage combinations. As a result, we determined that our tactile display can provide tactile feedback at $V_{pp}$ = 300 V and f = 5 Hz vibration.

### 3.3 Tactile feedback algorithm

Our method was designed to provide tactile feedback to the finger to evoke the sensation of complete detachment of the object from the skin. To achieve this, we opted to provide additional tactile feedback for a certain duration $\left(t_{1}+\Delta t\right)$ after the user lifts a finger off the physical object. The additional tactile feedback employed was a sine wave, a basic form of vibration. However, since the aim was to evoke the sensation of adhesion detachment from the skin, it was anticipated that continuous sinusoidal vibrations might inhibit this sensation. Therefore, this study used half-period sinusoidal vibrations rather than continuous sinusoidal vibrations. The tactile feedback was generated based on the applied voltage and frequency, which can be calculated using the following Equation 2.

$$V\left(t\right)=\left\{\begin{array}{cc} V_{pp}\sin\left(2\pi ft\right)\; & \text{if }0\leq t\leq \frac{1}{2f}\\ 0\; & \text{otherwise} \end{array}\right.$$ (2)

As the tactile feedback parameters varied between experiments, the specific $V_{pp}$ , f and displacement used in each experiment are detailed in their respective sections.

## 4 Experiment 1: Perceived adhesion evaluation

The primary objective of this experiment was to investigate whether participants’ perceived stickiness in adhesion-separation contact mode is augmented by providing additional tactile feedback at a certain duration after the finger lifts off from the object. To investigate this, we conducted an experiment varying the timing of the additional tactile feedback presentation.

### 4.1 Design and conditions

Participants were asked to touch a physical object twice while wearing our tactile display and to compare the two haptic stimuli. In one scenario, the haptic stimulus acted as the reference, producing only haptic sensations from the physical object without any additional tactile feedback from our tactile display. In the other scenario, the haptic stimulus served as a comparison, producing haptic stimulation from the physical object along with additional tactile feedback from our tactile display. After the two contact interactions, participants were tasked with comparing the two haptic stimuli they experienced and indicating which one exhibited stronger stickiness.

The physical object used in this experiment was consistent across all conditions. Additionally, the tactile feedback provided by our tactile display remained constant for all stimuli ( $V_{pp}$ = 325V, f = 20 Hz). This tactile stimulus was selected as the one with the highest perceived stickiness among the more than 30 stimuli explored in section 3.2. The output displacement of this stimulus was 26 μ m, based on the first-order linear approximation of voltage to displacement derived from the measurements described in section 3.2. However, the timing of this tactile feedback varied across conditions. Seven distinct presentation timings were employed:

• $t_{0}$ : This is the moment when the pressed force begins to decrease after the participant presses the physical object (Figure 1). In this experiment, t was defined as the instance when the pressed force drops below 1.5 N for the first time after the participant presses into the physical object and applies 1.5 N of pressure.

• $t_{1}$ : This is the moment when the force applied to the finger is closest to 0 N upon the separation of the finger from the physical object (Figure 1). In this experiment, t was defined as the point when the pressed force on the physical object first falls below 0.1 N after exceeding 1.5 N. The threshold value of 0.1 N was chosen instead of 0.0 N due to concerns about the possibility that the force might not drop to 0.0 N even after the participant releases their finger, owing to accumulated errors in the load cell during repeated interactions with the physical object.

• $t_{1}+0.1s$ : This is the moment 0.1s after the time when the force on the finger is closest to 0 N during the separation from the physical object.

• $t_{1}+0.2s$ : This is the moment 0.2s after the time when the force on the finger is closest to 0 N during the separation from the physical object.

• $t_{1}+0.3s$ : This is the moment 0.3s after the time when the force on the finger is closest to 0 N during the separation from the physical object.

• $t_{1}+0.4s$ : This is the moment 0.4s after the time when the force on the finger is closest to 0 N during the separation from the physical object.

• $t_{1}+0.5s$ : This is the moment 0.5s after the time when the force on the finger is closest to 0 N during the separation from the physical object.

Additionally, we conducted the experiment with a total of eight conditions, including one where no additional tactile feedback was provided. A total of 80 trials were conducted, with 10 trials for each condition. To mitigate any potential order effects between the reference stimulus (without additional tactile feedback) and the comparison stimulus (with additional tactile feedback), the presentation order of each was counterbalanced. The order of presentation for each condition was randomized.

### 4.2 Participants

Fifteen non-disabled participants (2 female, left-handed: 3, mean age: 24.3, SD: 2.74) participated in this experiment. The experimental protocol was approved by the Toyoda Gosei Co., Ltd. Ethics Committee. All participants provided informed consent and received compensation for their time.

### 4.3 Apparatus

**FIGURE 4:**
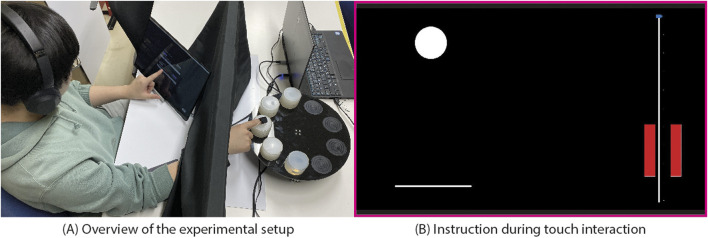
The setup of experiment. **(A)** overview of the experimental setup. **(B)** instruction during interaction.

**FIGURE 5:**
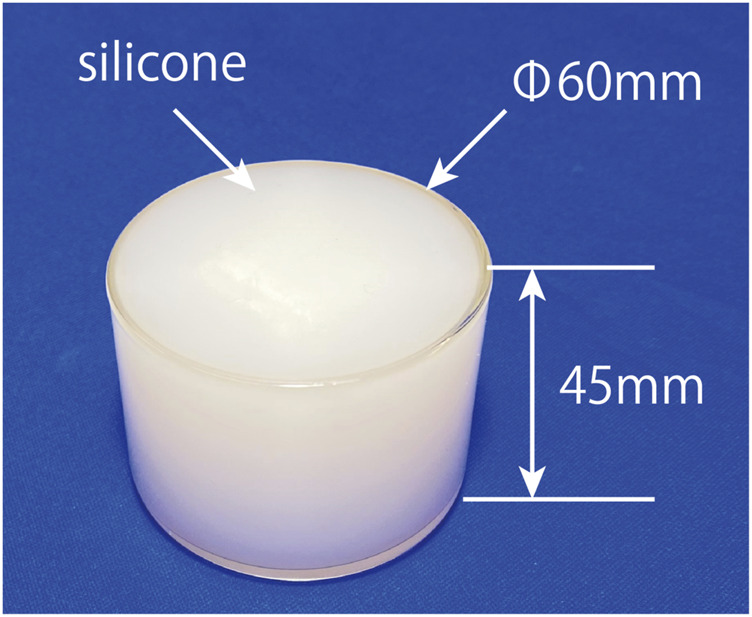
The Petri dish filled with silicone object.

The experimental setup is depicted in Figure 4. Participants wore our tactile display on the index finger of their right hand and were instructed to focus on a touch screen LCD displaying instructions. Adjacent to the LCD, a platform held a physical object, a cylindrical Petri dish filled with silicone, measuring 60 mm in diameter and 45 mm in height, with a Young’s modulus of 235 kPa (Figure 5). To mitigate potential bias from participants realizing they were touching the same object repeatedly, multiple physical objects were placed on the table. Additionally, a curtain was employed to obstruct the participant’s view of the table, preventing them from discerning which object they were touching. A calibrated load cell positioned beneath the table detected both the pressure exerted by the participant on the physical object and the moment when the participant’s finger lifted from it. To avoid interference from the driving noise of our tactile display, participants wore headphones emitting white noise throughout the experiment.

### 4.4 Procedure

Once the experiment commenced, participants were instructed to position their fingers above the physical object. Guided by instructions presented on the LCD, they proceeded to exert pressure on the object with their fingers. Subsequently, they lifted their fingers off from the object and returned them to the initial position. This sequence was repeated for a second contact interaction following cues displayed on the LCD. Following these two interactions, participants were prompted to complete a questionnaire comparing the two haptic stimuli they perceived. Upon questionnaire completion, the subsequent trial commenced, with participants repeating the same sequence until the experiment ended.

The pressing instruction displayed on the LCD comprised a white circle and a line (Figure 4B). At the start of the experiment, the circle descended to the line over a duration of 1 s, remained stationary on the line for 1 s, and then ascended for 1 s to return to its initial position. Participants were instructed to synchronize their finger movements with the circle’s motion to make contact with the physical object, utilizing the circle as a representation of their finger and the line as the surface of the physical object. Specifically, participants were directed to press down on the physical object with their finger for one second, maintain contact with the physical object for one second, and then retract their finger to its initial position by moving it away from the physical object for one second. This protocol was implemented to control the speed of finger movement and the duration of pressure application on the physical object. It was motivated by previous findings indicating that the duration of pressure application influences the perception of stickiness (Nam et al., 2020). Furthermore, given that finger pressure was showed to influence perceived stickiness (Nam et al., 2020), a pressure slider displayed on the LCD was used to control the pressure applied by the participants (Figure 4A). Participants were instructed to exert pressure on the physical object while monitoring the slider to control the pressure level.

The instructed pressure range was from 1.5 N, the minimum pressure utilized in (Nam et al., 2020), up to a maximum of 2.2 N. However, participants were permitted to exceed 2.2 N if maintaining a stable pressing force within this range proved challenging. This instruction aimed to reduce the participant’s load, allowing them to concentrate on evaluating the tactile sensation. In this experiment, participants were required to adjust the speed of finger movement, the duration of finger contact with the physical object, and the force applied while pressing the object, all according to the provided instructions. Due to the complexity of these tasks, participants were allowed to exceed a pressing force of 2.2 N if maintaining a stable force proved difficult.

After two contact interactions, participants were tasked with rating the perceived haptic stimuli on the following four items: Q1. The second haptic stimulus was stickier. Q2. How sticky was the second haptic stimulus? Q3. The second haptic stimulus was harder. Q4. How hard was the second haptic stimulus? Q1 and Q3 utilized a two-alternative forced choice format, where participants selected “yes” if they perceived the second haptic stimulus as stickier or harder, respectively. Otherwise, they chose “no.” Q2 and Q4 aimed to estimate the magnitude of perceived stickiness and hardness, with a response range from −10 to +10. Participants reported positive values if they perceived the second haptic stimulus as stickier or harder, and negative values if they perceived it as less sticky or softer. They could select 0 if they perceived the intensity between the two haptic stimuli as comparable. The stickiness questions in Q1 and Q2 were adapted from those used in (Lee et al., 2019). Meanwhile, the hardness questions in Q3 and Q4 were created by replacing stickiness with hardness. This addition of hardness questions aimed to detect any acquiescence bias among the participants. If only stickiness questions in Q1 and Q2 were used, participants could infer that the experimenter aimed to modulate stickiness with additional tactile feedback. Consequently, they could report feeling stickiness by acquiescence bias, even if they did not perceived stickiness by the additional tactile feedback. Thus, including questions about hardness, which were not expected to change with this experiment, provided a means to detect such bias. By analyzing responses to Q3 and Q4, any potential occurrence of acquiescence bias could be detected, allowing for a more comprehensive evaluation of the experiment’s outcomes. Finally, after completing all the trials, participants underwent semi-structured interviews addressing the question items (i.e., Q1 through Q4).

### 4.5 Results

**FIGURE 6:**
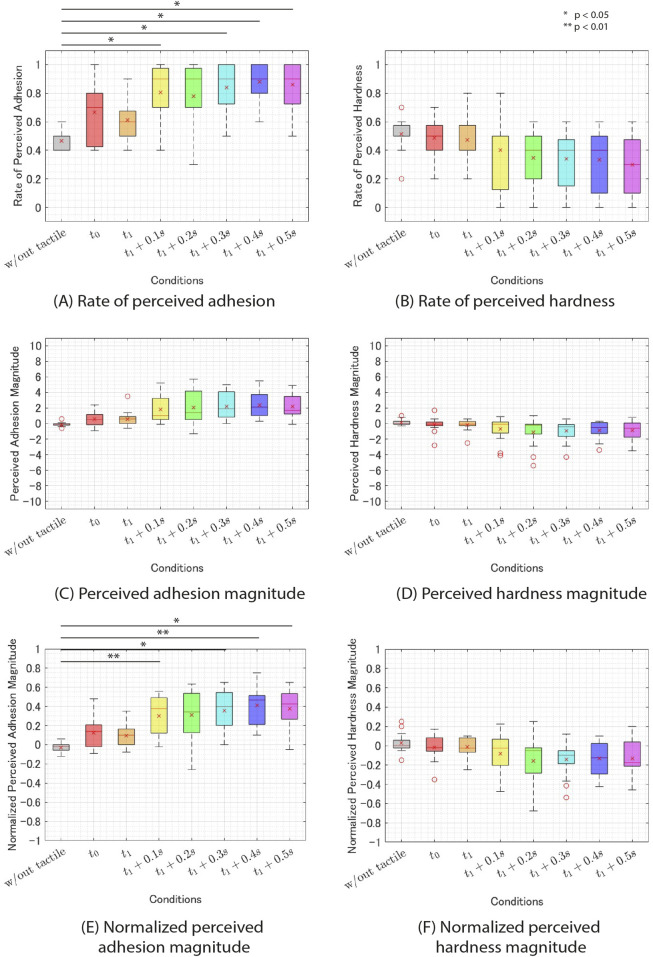
The results of experiment 1. **(A)** rate of perceived adhesion. **(B)** rate of perceived hardness. **(C)** perceived adhesion. **(D)** perceived hardness magnitude. **(E)** normalized percevied adhesion magnitude. **(F)** normalized percevied hardness magnitude.

Figure 6 shows the experimental results for each condition. Initially, a Shapiro-Wilk normality test was performed for the recognition rate of the stickier haptic stimulus (Q1) and the recognition rate of the harder stimulus (Q3), indicating a violation of the normality assumption for both data. Consequently, a Wilcoxon signed-rank test with Bonferroni correction was employed. The analysis revealed a significant difference in the recognition rate of the sticker haptic stimulus (Q1) between the condition without additional tactile feedback and the $t_{1}+0.1s$ , $t_{1}+0.3s$ , $t_{1}+0.4s$ , and $t_{1}+0.5s$ conditions ( p=0.03 , 0.019, 0.018, and 0.019, respectively). Conversely, no significant difference was observed between conditions for the recognition rate of the harder stimulus (Q2).

Next, we analyzed the perceived magnitude of stickiness (Q2) and hardness (Q4). Since the maximum and minimum values of perceived stickiness and hardness varied across participants, the results were normalized by each participant’s maximum absolute response value. The normalized results are presented in Figures 6E, F. A Shapiro-Wilk normality test was conducted on the two sets of normalized results, revealing that both were normally distributed. However, Mendoza’s multisample sphericity test indicated that the normalized perceived stickiness magnitude violated the assumption of sphericity. Therefore, we performed a Wilcoxon signed-rank test with Greenhouse-Geisser adjustment and Bonferroni correction. The results revealed a significant difference in normalized perceived stickiness magnitude between the condition without additional tactile feedback and the $t_{1}+0.1s$ , $t_{1}+0.3s$ , $t_{1}+0.4s$ , and $t_{1}+0.5s$ conditions ( p<0.01,p=0.020,p<0.01,p=0.020 , respectively). Conversely, no significant difference was observed among conditions for normalized perceived hardness magnitude.

### 4.6 Discussion

#### 4.6.1 The recognition rate of stickier haptic stimulus

The experimental results for the recognition rate of the stickier haptic stimulus (Q1) revealed that the $t_{1}+0.1s$ , $t_{1}+0.3s$ , $t_{1}+0.4s$ , and $t_{1}+0.5s$ conditions, where additional tactile feedback was presented at certain time after the finger lifted off from the physical object, led to significantly higher perceived stickiness compared to the condition without additional tactile feedback. This finding was supported by the fact that approximately half of the participants (p5, p7, p10, p11, p13, p14, p15) reported perceiving a difference in stickiness during post-experimental interviews. These results suggest that perceived stickiness can be augmented by presenting additional tactile feedback at the appropriate time after the finger lift off the physical object. However, it should be noted that the results of this experiment may not have been obtained under a constant pressure of from 1.5 to 2.2 N. We allowed the pressing force to exceed 2.2 N in cases where maintaining a stable force was challenging, as the manipulation required was not easy. Consequently, some trials may not have perceived additional tactile feedback due to excessive pressing force. Alternatively, even if additional tactile feedback was perceived, there may have been trials where this feedback was not associated with stickiness due to an unnatural balance between the intensity of the vibration and the pressing force. Therefore, investigating the limits of the pressing force for which this method remains effective is a important consideration for designing practical applications.

The displacement of the tactile feedback used to validate this method and the force applied to the finger were only 26.4 μ m and 14.0 mN, respectively. Moreover, this method lacks the continuous tensile force that would typically occur when a real adherent object is attached to the finger, and instead, discontinuous tactile feedback is presented as the tactile stimulus. Despite these limitations, participants reported a perceived change in the stickiness of the physical object. This result suggests the potential to augment perceived stickiness without the need for a bulky haptic display to generate physically accurate adhesiveness or suction. Consequently, this method is expected to be particularly useful for presenting adhesion in applications that require thin and soft tactile displays, such as HAR and wearable.

#### 4.6.2 The recognition rate of the harder haptic stimulus

The experimental results for the recognition rate of the harder haptic stimulus (Q3) showed no significant differences between conditions. During post-experiment interviews, some participants (p4, p6, p10, p14, p15) reported little or no perception of differences in hardness. These results align with our expectation that presenting additional tactile feedback when lifting the finger off from the physical object does not alter the perceived hardness. Simultaneously, this outcome dismisses the possibility that the perceived stickiness results obtained in this experiment were influenced by acquiescence bias, thereby increasing the certainty of our analysis that our proposed method augments perceived stickiness.

However, contrary to our expectations, some subjects (p2, p9) reported perceiving more differences in hardness than in stickiness. Another subject (p8) reported that changes in stickiness also influenced the sensation of hardness. To further examine these results, let us consider objects with varying hardness. A hard object requires a strong force for elongation, while a soft object requires a weaker force. Consequently, if both objects possess the same adhesive strength, the hard object would detach the adhesive from the finger shortly after removal, whereas the soft object would take a comparatively longer time for complete detachment. The manipulation of the timing of additional tactile feedback presentation in this experiment may have been perceived as differences in detachment timing due to variations in hardness, thereby participants may have recognized differences in hardness. Further investigation into the relationship between stickiness and hardness sensation could contribute to the development of new haptic presentation techniques and a deeper understanding of human haptic perception mechanisms.

#### 4.6.3 Perceived adhesion magnitude

The results of the experiment on normalized perceived stickiness magnitude (Q2) indicated that the conditions $t_{1}+0.1s$ , $t_{1}+0.3s$ , $t_{1}+0.4$ s, and $t_{1}+0.5$ s exhibited significantly higher perceived stickiness compared to the condition without additional tactile feedback. However, as depicted in Figure 6, the intensity of perceived stickiness was relatively low, averaging only around +2 , whereas the maximum value on the scale was set at +10 . These results suggest that although the additional tactile feedback presented in this experiment could augment the perceived stickiness, it might have resulted in a weak rather than a strong perception of stickiness. Indeed, during a post-experiment participant interview, one participant (p10) described the sensation elicited by the tactile feedback as a mild stickiness, similar to peeling off double-sided tape.

In the $t_{1}+0.1s$ , $t_{1}+0.3s$ , $t_{1}+0.4s$ , and $t_{1}+0.5s$ conditions, no significant correlation was observed between the duration t and the perceived stickiness intensity. Contrary to our expectations based on prior research indicating a correlation between the duration of time until the finger detaches from the physical object and perceived stickiness intensity, correlation between t and perceived stickiness intensity was not supported by the experimental results.

One possible reason for this result could be attributed to the effect of the small displacement of the additional tactile feedback utilized in this experiment. As noted in Section 4.6.1, the expected displacement and force of the tactile feedback employed in this experiment were only 26.4 μm and 14.0 mN, respectively. Consequently, the perceived adhesive strength from this small skin deformation may have been constrained, potentially resulting in the inability to perceive a strong adhesive force.

Another potential factor is the absence of continuous tension exerted on the finger by the tactile feedback in our method. When interacting with a physically sticky object and subsequently releasing the finger, continuous tension and impulse are exerted on the finger by the adhesive. (Nam et al., 2020). reported that this impulse also correlates with perceived stickiness intensity. However, our approach is unable to reproduce this continuous tension and the impulse exerted by it. Hence, the absence of these factors may account for the weak adhesion perceived with our approach and the lack of correlation between t and perceived adhesion. Further exploration is needed to explore methods for enhancing the perceived adhesion strength using our approach.

## 5 Experiment 2: Evaluation of haptic experience

Our proposed stickiness augmentation approach for adhesion-separation contact mode in HAR involves presenting additional tactile feedback after a certain time after the user lifts a finger off the physical object. This approach differs from real-life adhesion phenomena, where the finger adheres to a physical object, and does not reproduce the continuous tension experienced between the finger and the adhering object when attempting to detach. Consequently, the stickiness experience generated by our method is not typical of everyday experiences. Hence, it is crucial to gain a deeper understanding of how users perceive the stickiness experience generated by our method to appropriately design applications using our approach. For instance, will users like it? Will the additional tactile feedback harmonize with the haptic stimuli obtained from the physical object? Do differences in the timing of additional tactile feedback presentation affect the stickiness experience? Do differences in the frequency of additional tactile feedback influence perceived stickiness or realism? Is there an interaction between presentation timing and frequency? In this experiment, we conducted a subjective evaluation of the stickiness sensation experience generated by our method to address some of these questions.

### 5.1 Design and conditions

Participants were instructed to touch a physical object to perceive the haptic sensation and then evaluate their haptic experience. In this experiment, following the protocol of Section 4, participants pressed their fingers onto the physical object in response to the movement of a circle displayed on the LCD screen, and subsequently lifted their fingers from the object. Additional tactile feedback was presented through our tactile display after the removal of the finger from the physical object. Subsequently, participants completed a questionnaire to evaluate the tactile feedback they experienced. Unlike in section 4, participants in this experiment encountered a single haptic feedback and provided subjective ratings solely for that experience. Moreover, participants had the opportunity to experience the haptic sensation multiple times until they were satisfied.

The additional tactile feedback generated by our tactile display can be determined using Equation 2. In this experiment, five frequencies of additional tactile feedback were utilized: f = 5, 10, 20, 70, and 250 Hz. These frequencies were selected based on the sensitivity of SAI, RAI, and RAII mechanoreceptors (Bolanowski et al., 1988), as well as the intermediate value of each frequency when log-transformed. Since the perceptual process in our approach is not clear, these frequencies were chosen to broadly stimulate the mechanoreceptors. The peak-to-peak voltage Vpp of the additional tactile feedback was adjusted to yield the same output displacement at each frequency. Specifically, 32 μm was first selected as the output displacement at which vibration could be perceived from the exploration for more than 30 vibration patterns conducted in Section 3.2. Next, from the measurement results in Section 3.2, linear first-order approximation equations for the applied voltage versus output displacement at each frequency were derived, and the applied voltage required for an output of 32 μm was calculated at each frequency. As a result, the corresponding applied voltages at each frequency were determined to be $V_{pp,5Hz}$ = 0.29, $V_{pp,10Hz}$ = 0.32, $V_{pp,20Hz}$ = 0.35, $V_{pp,70Hz}$ = 0.34, $V_{pp,250Hz}$ = 0.33 kV, respectively. The timing for presenting the additional tactile feedback included three conditions used in Section 4: $t_{1}+0.1s$ , $t_{1}+0.3s$ , and $t_{1}+0.5s$ . The experiment comprised a total of 15 conditions, obtained by combining the 5 frequencies and 3 presentation timings. A total of 30 trials were conducted, with two trials for each condition. The presentation order of each condition was randomized.

### 5.2 Participants

Ten non-disabled participants (2 female, mean age: 23.5, SD: 1.63) participated in this experiment. Eight of these participants were the same as those in Experiment 1. The experimental protocol was approved by the Toyoda Gosei Co., Ltd. Ethics Committee. All participants provided informed consent and received compensation for their time.

### 5.3 Apparatus

The experimental setup was replicated in Section 4. Participants were instructed to wear our tactile display on the index finger of their right hand and to focus on a touchscreen LCD displaying instructions. Adjacent to the LCD, a load cell equipped with a platform holding a physical object was positioned and concealed from the participants’ view by a curtain. Additionally, to avoid auditory distractions, participants wore headphones emitting white noise.

### 5.4 Procedure

Participants were required to experience additional tactile feedback generated at five different frequencies and three different presentation timings to respond to the questionnaire. Initially, participants were instructed to touch a physical object according to the instructions on the LCD touch panel. Participants were allowed to touch the physical object as many times as they wished until they were satisfied. After touching the physical object, they were required to evaluate their experience on the following 7 items: Q1. I felt adhesive due to the haptic feedback, Q2. I felt hardness due to the haptic feedback, Q3. The haptic feedback was realistic, Q4. The haptic feedback was believable, Q5. The haptic feedback was convincing, Q6. The haptic feedback was pleasant, Q7. The haptic feedback felt out of place. Q1 and Q2 were questions about stickiness and hardness, respectively. Q3, Q4, and Q5 were items to assess the realism of the haptic experience obtained, using questions proposed by (Sathiyamurthy et al., 2021). Q6 evaluated the pleasantness or unpleasantness of the haptic experience obtained, adopting questions used by (Bau et al., 2010). Q7 evaluated the harmony between the additional tactile feedback and the haptic stimuli obtained from the physical object, using questions proposed by (Sathiyamurthy et al., 2021). Participants rated these seven question items using a 7-point Likert scale. A score of 1 indicated “strongly disagree,” while 7 indicated “strongly agree.”

Participants were instructed to select a rating of 1 on all items if they did not perceive any additional tactile feedback after a number of touches. While it is common practice to instruct subjects to select 4, representing a neutral response, in this experiment, they were instructed to select 1 of all items. This instruction aimed to distinguish between instances where participants did not perceive additional tactile feedback and instances where participants selected 4 for all items. In this experiment, Q1 through Q6 represent the positive items in the questionnaire, with a rating of 7 indicating the most positive evaluation. While Q7 represents the negative item, with a rating of 7 indicating the most negative evaluation. Consequently, while it was possible for ratings from Q1 to Q7 to all become 4, it was considered unlikely for all of them to become 1. Thus, it was believed that by selecting 1 for all items, trials where additional tactile feedback was not perceived could be detected. However, since all items rated as 1 introduce bias in the results when analyzing the experimental data, after extracting the trials where additional tactile feedback was not perceived, and the results of these trials were discarded in the subsequent analysis. Finally, after completing all the trials, participants underwent semi-structured interviews addressing the question items (i.e., Q1 through Q7)

### 5.5 Results

**FIGURE 7:**
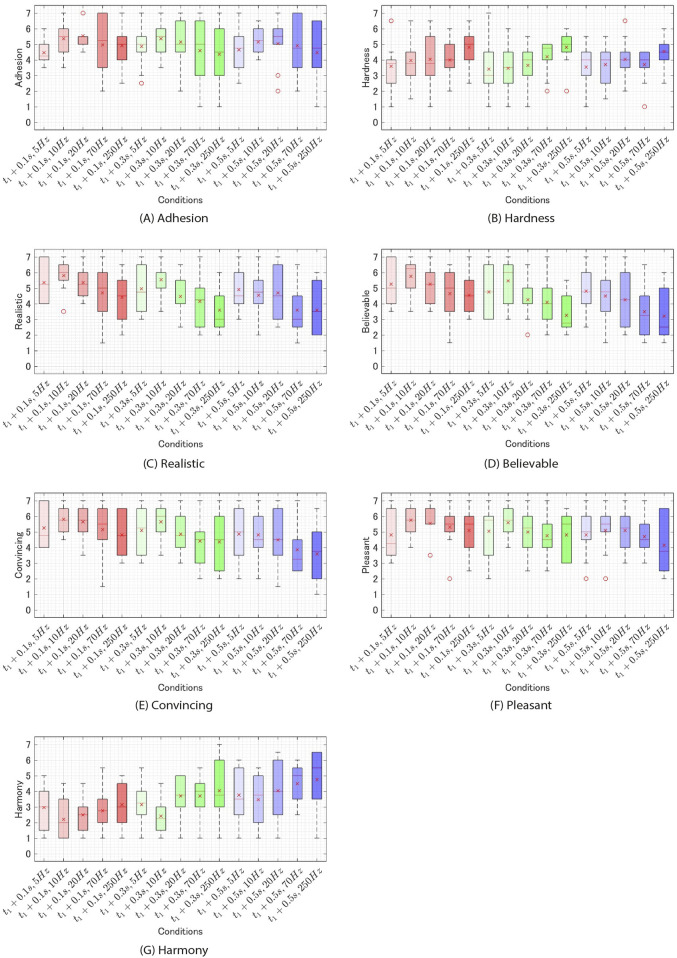
The results of experiment 2 for each condition. **(A)** adhesion. **(B)** hardness. **(C)** realistic. **(D)** believable. **(E)** convincing. **(F)**. pleasant. **(G)**. harmony.

In conditions of f=5 Hz, $t_{1}+0.1s$ , additional tactile feedback was not perceived in 7 out of 20 trials (35%) . In conditions of f=5 Hz, $t_{1}+0.3s$ , and in conditions of f=250 Hz, $t_{1}+0.1s$ , additional tactile feedback was not perceived in 1 out of 20 trials (5%) . Therefore, the results of these trials were discarded. The experimental results for each condition are depicted in Figure 7. Initially, a Shapiro-Wilk normality test was performed on each question item, indicating a violation of the normality assumption for all questions. Subsequently, a two-way ANOVA with the Aligned Rank Transform (Wobbrock et al., 2011) was conducted. The results revealed a main effect of timing for Q3, Q4, Q5, Q6, and Q7. (Q3: F(2,276)=7.89,p<0.01 , Q4: F(2,276)=9.89,p<0.01 , Q5: F(2,276)=8.99,p<0.01 , Q6: F(2,276)=4.68,p=0.010 , Q7: F(2,276)=15.40,p<0.01 ). Additionally, a main effect of frequency was observed for Q2, Q3, Q4, Q5, and Q7. (Q2: F(4,276)=5.50,p<0.01 , Q3: F(4,276)=8.62,p<0.01 , Q4: F(4,276)=8.08,p<0.01 , Q5: F(4,276)=3.92,p<0.01 , Q7: F(4,276)=3.71,p<0.01 ). Conversely, there was no interaction between timing and frequency across all questions.

**FIGURE 8:**
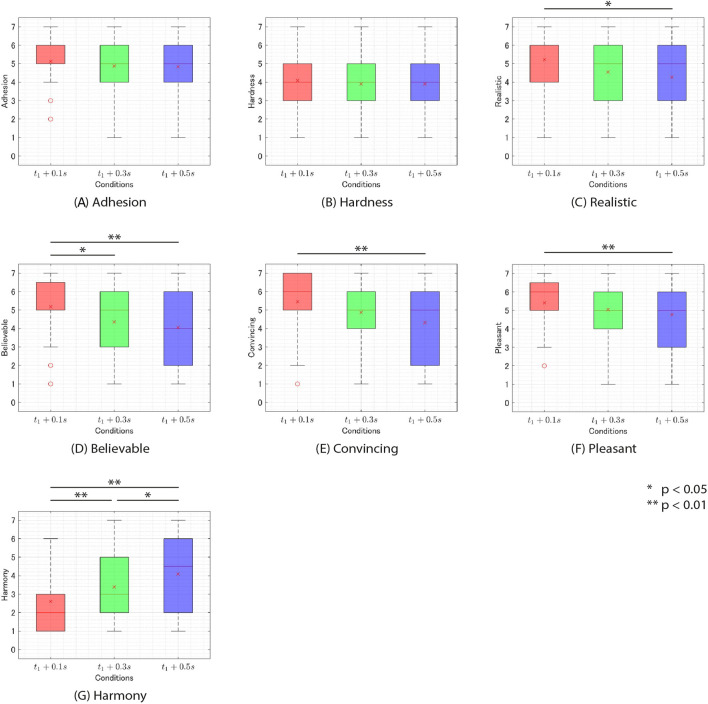
The rating of questionnaire on experiment 2 v. s. feedback timing of additional tactile feedback. **(A)** adhesion. **(B)** hardness. **(C)** realistic. **(D)** believable. **(E)** convincing. **(F)**. pleasant. **(G)**. harmony.

**FIGURE 9:**
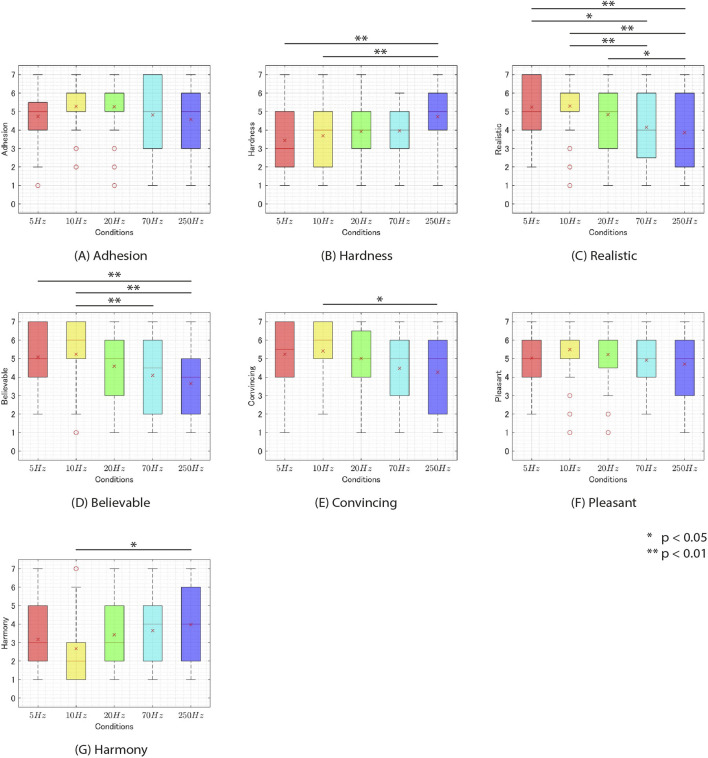
The rating of questionnaire on experiment 2 v. s. frequency of additional tactile feedback. **(A)** adhesion. **(B)** hardness. **(C)** realistic. **(D)** believable. **(E)** convincing. **(F)**. pleasant. **(G)**. harmony.

Next, for the questions where a main effect of timing was observed (Q3-Q7) and for those where a main effect of frequency was observed (Q2-Q7), a Wilcoxon signed-rank test with Bonferroni correction was conducted. The results revealed significant differences between the conditions as shown in Table 1 (Figures 8, 9).

**TABLE 1:** Combinations of haptic stimuli with significant differences indicated by multiple comparisons.

Question	Timing [s]	Timing [s]	p
Q3	0.1	0.5	=.024
Q4	0.1	0.3	=.018
	0.1	0.5	<.01
Q5	0.1	0.5	<.01
Q6	0.1	0.5	<.01
Q7	0.1	0.3	<.01
	0.1	0.5	<.01
	0.3	0.5	=.030

Question	f [Hz]	f [Hz]	p
Q2	5	250	<.01
	10	250	<.01
Q3	5	70	=.018
	5	250	<.01
	10	70	<.01
	10	250	<.01
	20	250	=.031
Q4	5	250	<.01
	10	70	<.01
	10	250	<.01
Q5	10	250	=.026
Q7	10	250	=.012

### 5.6 Discussion

#### 5.6.1 Stickiness

First, the t1+0.1s and f = 20 Hz condition produced the highest score of 5.6. This score falls between “somewhat agree” (5) and “agree” (6), suggesting that subjects may have perceived stickiness on the t1+0.1s and f = 20 Hz condition. Next, A two-way ANOVA with ART correction showed no main effects of frequency and timing or interaction. This result suggests that there were no significant differences between the aforementioned t1+0.1s and f = 20 Hz condition and other stimuli. Therefore, it is possible that stickiness was perceived in all conditions used in this experiment similarly to the t1+0.1s and f = 20 Hz condition. However, the t1+0.3s and f = 70 Hz condition, which had the lowest score, produced a score of 4.4. This score is higher than “strongly disagree” (1) and “neutral” (4) but closer to “neutral” (4) than to “strongly agree” (7), indicating that the stickiness was not clearly perceived. Therefore, it would be appropriate to conduct additional research to determine whether participants could perceive stickiness even under low-scoring conditions such as the t1+0.3s and f = 70 Hz condition.

Moreover, in the $t_{1}+0.1s$ , f = 5 Hz condition, approximately half of the respondents (7 out of 20 trials, 35% ) reported not perceiving additional tactile feedback. The number of times that additional tactile feedback was not perceived in the other conditions was 1 out of 20 (5%) in both the $t_{1}+0.1s$ and f=5 Hz, and $t_{1}+0.3s$ and f=5 Hz conditions. Thus, the reason why the additional tactile feedback was not perceived in conditions with short latency or low frequency could be due to the masking effect of the haptic feedback from the physical object. For instance, in the low-frequency band such as f=5 Hz, the perceptual threshold for tactile stimuli is higher compared to the high-frequency band, making it more difficult to perceive vibrations. Additionally, in the $t_{1}+0.1s$ condition, tactile feedback might have been presented immediately after the finger was lift off from the physical object, causing it to confuse with the haptic feedback from the physical object. This confusing could lead to the additional tactile feedback being perceived as part of the physical object’s haptic feedback, rather than as separate additional tactile feedback. Therefore, in the $t_{1}+0.1s$ condition with small latency and the f=5 Hz condition with low-frequency vibration, the additional tactile feedback was likely masked by the haptic feedback from the physical object, making it less perceivable. Therefore, when using a haptic display with small displacement to augment stickiness using this method, it is desirable to use vibrations at frequencies with lower perceptual thresholds or to increase the touch presentation timing Δt to reduce the masking of additional tactile feedback by haptic stimuli from the physical object.

#### 5.6.2 Hardness

Significant differences were observed between the 250 Hz frequency and 5 Hz and between 250Hz and 10 Hz regarding the sensation of hardness in Q2 (Figure 9B). This suggests that lower frequencies of additional tactile feedback made the physical object feel softer, while higher frequencies made the physical object feel harder.

The result is not surprising since, when considering two objects with the same adhesive strength but differing hardness adhering to a finger, it is expected that when the finger detaches from the objects at a constant speed, the softer object will detach more slowly than the harder one. However, it is essential to consider these results alongside the finding that differences in frequency do not affect the perceived stickiness (Q1). This is important because these results suggest the potential to independently control the perceived hardness without altering the perceived stickiness by adjusting the frequency of additional tactile feedback. This outcome implies the existence of optimal parameters for additional tactile feedback for augmentation stickiness based on the hardness of the object. For example, using higher-frequency vibrations around 250 Hz may be ideal for modulating the adhesive sensation of harder objects, while lower frequencies around 10 Hz might work better for softer objects. Investigating methods to design appropriate tactile feedback for augmentation stickiness based on the desired tactile sensation is crucial for realizing applications using this method.

#### 5.6.3 Realism

Q3, Q4, and Q5 were all questions regarding realism. These questions showed significant main effects of both timing and frequency. As depicted in Figures 8C–E, presenting additional tactile feedback at lower Δt values, such as 0.1s, resulted in significantly higher realism compared to 0.3s and 0.5s. This result can be attributed to the inability of our method to present the continuous tension and impulse exerted on the finger when separating from the adhered object. Consequently, as the value of Δt increases, the disparity from the physical stickiness phenomenon may become more pronounced, leading to discomfort. However, as mentioned in Section 5.6.1, if Δt is too small, there is a risk that the additional tactile feedback may be masked by the haptic stimuli obtained from the physical object, potentially resulting in the loss of perception of the additional tactile feedback. Therefore, Δt needs to be appropriately designed based on the available tactile feedback frequencies and their intensity.

Next, the condition with low-frequency band vibration exhibited significantly higher realism compared to conditions with high-frequency band vibration (Figures 9C–E). This observation could be attributed to the fact that low-frequency vibrations, which evoke the sensation of softness (Section 5.6.2), aligned with users’ expectations that an object taking a certain time to detach from the finger is soft and prone to deformation. Conversely, the absence of a significant difference for the condition with f = 5Hz, lower than 10Hz, compared to other frequencies might be attributed to the fact that additional tactile feedback was not perceived in some instances under the $t_{1}+0.1s$ condition for 5 Hz.

#### 5.6.4 Pleasant

For pleasantness (Q6), the main effect was observed for timing. The $t_{1}+0.1s$ condition was significantly more pleasant than the $t_{1}+0.5s$ condition for presentation timing (Figure 8F). This could be attributed to the higher realism, which generally might lead to a more enjoyable experience for the participants. However, even in the least pleasant $t_{1}+0.5s$ condition, the mean rating was 4.8, surpassing the neutral rating of 4.0. This value, although representing the lowest level of pleasantness, still indicates a pleasant experience, suggesting an overall pleasant haptic experience. Indeed, during post-experiment interviews, more than half of the participants (p1, p2, p4, p6, p7, p9, and p10) reported an overall lack of unpleasantness.

#### 5.6.5 Harmony

For Harmony (Q7), significant main effects were observed for both frequency and timing. Regarding timing, notable differences were observed across all conditions (Figure 8G). The $t_{1}+0.1s$ condition exhibited the highest level of harmony, indicating consistency with both the haptic feedback from the physical object and the additional tactile feedback. Conversely, the later the presentation of the additional tactile feedback, the less harmonious it appeared. This disparity may be attributed to the method’s inability, akin to the Realism (Q3, Q4, and Q5) aspect, to present the continuous tension or impulse when the finger interacts with the object. As the timing Δt is delayed, the discontinuity between the haptic feedback from the physical object and the additional tactile feedback becomes more pronounced, potentially reducing the sense of harmony.

In Figure 9G, the f = 10 Hz condition showed significantly higher harmony compared to the f = 70 Hz and 250 Hz conditions. This outcome, similar to the results of Realism (Q3, Q4, and Q5), could be attributed to the fact that low-frequency vibrations, which evoke the sensation of softness (Section 5.6.2), aligned with users’ expectations that an object taking a certain time to detach from the finger is soft and prone to deformation.

## 6 Conclusion

In this study, we introduced a novel approach for HAR that augments the perceived stickiness of a physical object during adhesion-separation contact mode by presenting additional tactile feedback after a particular time after the finger lifts off from the physical object. To validate this approach, we developed a DEA-based thin-film, soft wearable tactile display tailored for HAR applications. Through two user experiments, we assessed the effectiveness of our method. In Experiment 1, we investigated the modulation of perceived stickiness by varying the presentation timing of additional tactile feedback. Our findings indicate that presenting additional tactile feedback after a particular time after the finger lifts off augments perceived stickiness. In Experiment 2, we further explored the stickiness experience by testing various frequencies and presenting timings of additional tactile feedback. While stickiness was perceived across all feedback conditions, the realism and harmony of the experience were influenced by the frequency and presentation timing of the additional tactile feedback.

Although this approach was initially designed for HAR, it could be adapted to VR and AR, it may hold promise for a diverse array of applications. We believe that our method will enrich haptic experiences in these domains, contributing to the advancement of haptic technology and the development of innovative applications.

## Data Availability

The raw data supporting the conclusions of this article will be made available by the authors, without undue reservation.
